# Identification and characterization of the TCA cycle genes in maize

**DOI:** 10.1186/s12870-019-2213-0

**Published:** 2019-12-27

**Authors:** Yongming Liu, Jingtao Qu, Ling Zhang, Xiangyu Xu, Gui Wei, Zhuofan Zhao, Maozhi Ren, Moju Cao

**Affiliations:** 1 Institute of Urban Agriculture, Chinese Academy of Agricultural Sciences, Chengdu, 610213 China; 2 Chengdu National Agricultural Science and Technology Center, Chengdu, 610213 China; 3 Key Laboratory of Biology and Genetic Improvement of Maize in Southwest Region of Ministry of Agriculture, Maize Research Institute, Sichuan Agricultural University, Chengdu, 611130 China; 4 Department of Plant Biotechnology and Bioinformatics, Ghent University, B-9052 Ghent, Belgium; 5 VIB Center for Plant Systems Biology, B-9052 Ghent, Belgium; 6 College of Agronomy, Sichuan Agricultural University, Chengdu, 611130 China

**Keywords:** Maize, TCA cycle, Root development, Salt stress, Plant fertility

## Abstract

**Background:**

The tricarboxylic acid (TCA) cycle is crucial for cellular energy metabolism and carbon skeleton supply. However, the detailed functions of the maize TCA cycle genes remain unclear.

**Results:**

In this study, 91 TCA genes were identified in maize by a homology search, and they were distributed on 10 chromosomes and 1 contig. Phylogenetic results showed that almost all maize TCA genes could be classified into eight major clades according to their enzyme families. Sequence alignment revealed that several genes in the same subunit shared high protein sequence similarity. The results of cis-acting element analysis suggested that several TCA genes might be involved in signal transduction and plant growth. Expression profile analysis showed that many maize TCA cycle genes were expressed in specific tissues, and replicate genes always shared similar expression patterns. Moreover, qPCR analysis revealed that some TCA genes were highly expressed in the anthers at the microspore meiosis phase. In addition, we predicted the potential interaction networks among the maize TCA genes. Next, we cloned five TCA genes located on different TCA enzyme complexes, Zm00001d008244 (isocitrate dehydrogenase, IDH), Zm00001d017258 (succinyl-CoA synthetase, SCoAL), Zm00001d025258 (α-ketoglutarate dehydrogenase, αKGDH), Zm00001d027558 (aconitase, ACO) and Zm00001d044042 (malate dehydrogenase, MDH). Confocal observation showed that their protein products were mainly localized to the mitochondria; however, Zm00001d025258 and Zm00001d027558 were also distributed in the nucleus, and Zm00001d017258 and Zm00001d044042 were also located in other unknown positions in the cytoplasm. Through the bimolecular fluorescent complimentary (BiFC) method, it was determined that Zm00001d027558 and Zm00001d044042 could form homologous dimers, and both homologous dimers were mainly distributed in the mitochondria. However, no heterodimers were detected between these five genes. Finally, Arabidopsis lines overexpressing the above five genes were constructed, and those transgenic lines exhibited altered primary root length, salt tolerance, and fertility.

**Conclusion:**

Sequence compositions, duplication patterns, phylogenetic relationships, cis-elements, expression patterns, and interaction networks were investigated for all maize TCA cycle genes. Five maize TCA genes were overexpressed in Arabidopsis, and they could alter primary root length, salt tolerance, and fertility. In conclusion, our findings may help to reveal the molecular function of the TCA genes in maize.

## Background

The tricarboxylic acid (TCA) cycle, which is also known as the Krebs cycle or the citric acid cycle, was discovered by Hans Krebs in 1937. The TCA cycle is ubiquitous in animals, plants and microbial cells and is crucial for cellular energy and carbon skeleton supply, especially for sugar catabolism, fat catabolism, and protein catabolism [1]. In general, the TCA cycle consists of eight enzymes, citrate synthase (CSY), aconitase (ACO), isocitrate dehydrogenase (IDH), α-ketoglutarate dehydrogenase complex (αKGDHC), succinyl-CoA synthetase (SCoAL), succinate dehydrogenase (SDH), fumarase (FUM), and malate dehydrogenase (MDH). A series of studies on TCA mutants in tomato showed that fumarase, malate dehydrogenase, and ɑ-ketoglutarate dehydrogenase play key roles in regulating the metabolic level of the TCA cycle [2]. Moreover, most of the reactions of the TCA cycle are reversible, except for the synthesis of citric acid and succinyl-CoA [3, 4]. In addition, the reactions catalysed by SCoAL and SDH in the TCA cycle can only be carried out in mitochondria, while other reactions in the TCA cycle can be replaced by similar reactions in other subcellular compartments [2].

The TCA cycle genes are associated with root and cotyledon development [5, 6], leaf senescence [7], flower development [8–10], fruit ripening [7, 11], seed germination [12, 13] and other developmental processes [3, 14]. The molecular function of most tomato TCA enzyme complexes has been investigated in detail [2]. For example, the inhibition of malate dehydrogenase, aconitase or fumarase could reduce respiratory activity and dry matter accumulation in roots and increase fruit yield. However, the photosynthetic properties of tomato leaves were improved in the malate dehydrogenase and aconitase antisense mutants but decreased in the fumarase antisense mutants [15–18]. Strangely, the mutants did not exhibit any significant changes when citrate synthase, succinyl-CoA synthetase, or isocitrate dehydrogenase was inhibited [19]. This is probably because most of the reactions in the TCA cycle could be replaced by similar reactions in other subcellular locations [20].

In addition to supplying energy, the TCA cycle participates in many metabolic pathways in cells, including photosynthesis [19, 21], photorespiration [22], abiotic stress [23–27], circadian clock [28], hormone signalling [29], and glycolysis [30]. To date, many factors that directly interact with TCA proteins or regulate cellular TCA cycle activity have also been identified. For example, malate dehydrogenase in the TCA cycle could interact with phosphoenolpyruvate carboxykinase in gluconeogenesis, indicating a direct link between the TCA cycle and gluconeogenesis [31]. Moreover, 125 interactions between the TCA cycle enzyme subunits and proteins associated with other pathways (mitochondrial electron transport complex/ATP synthesis, amino acid metabolism, and redox stress) have been identified [14]. The transcription factor bZIP14 in the diatom *Phaeodactylum tricornutum* could bind the promoters of TCA genes and directly activate TCA cycle gene expression [27]. In addition, thioredoxin has been shown to regulate the activity of the mitochondrial TCA cycle by modulating the thiol redox status [32]. In summary, the above results reflect the diversity and complexity of TCA cycle functions.

The TCA cycle is critical for cellular energy metabolism and plays a key role in many developmental processes; therefore, uncovering the molecular function of TCA cycle genes may provide deeper insights into plant development. To date, most of the experiments on TCA cycle genes have been conducted in Arabidopsis and tomato. In maize, the TCA metabolic level is closely related to root development [33], phosphorus deficiency [34], and drought and salt stresses [35–37], but the molecular functions of TCA genes remain unclear. In this study, we identified all the TCA cycle genes in maize and analysed their phylogenetic relationships and expression patterns. Subsequently, their cis-acting elements, subcellular localizations, and interaction networks were analysed. Finally, five TCA genes were respectively overexpressed in Arabidopsis, and their effects on root growth, salt-stress resistance, and reproductive growth were examined.

## Results

### Bioinformatics analysis of the maize TCA cycle genes

**Fig. 1:**
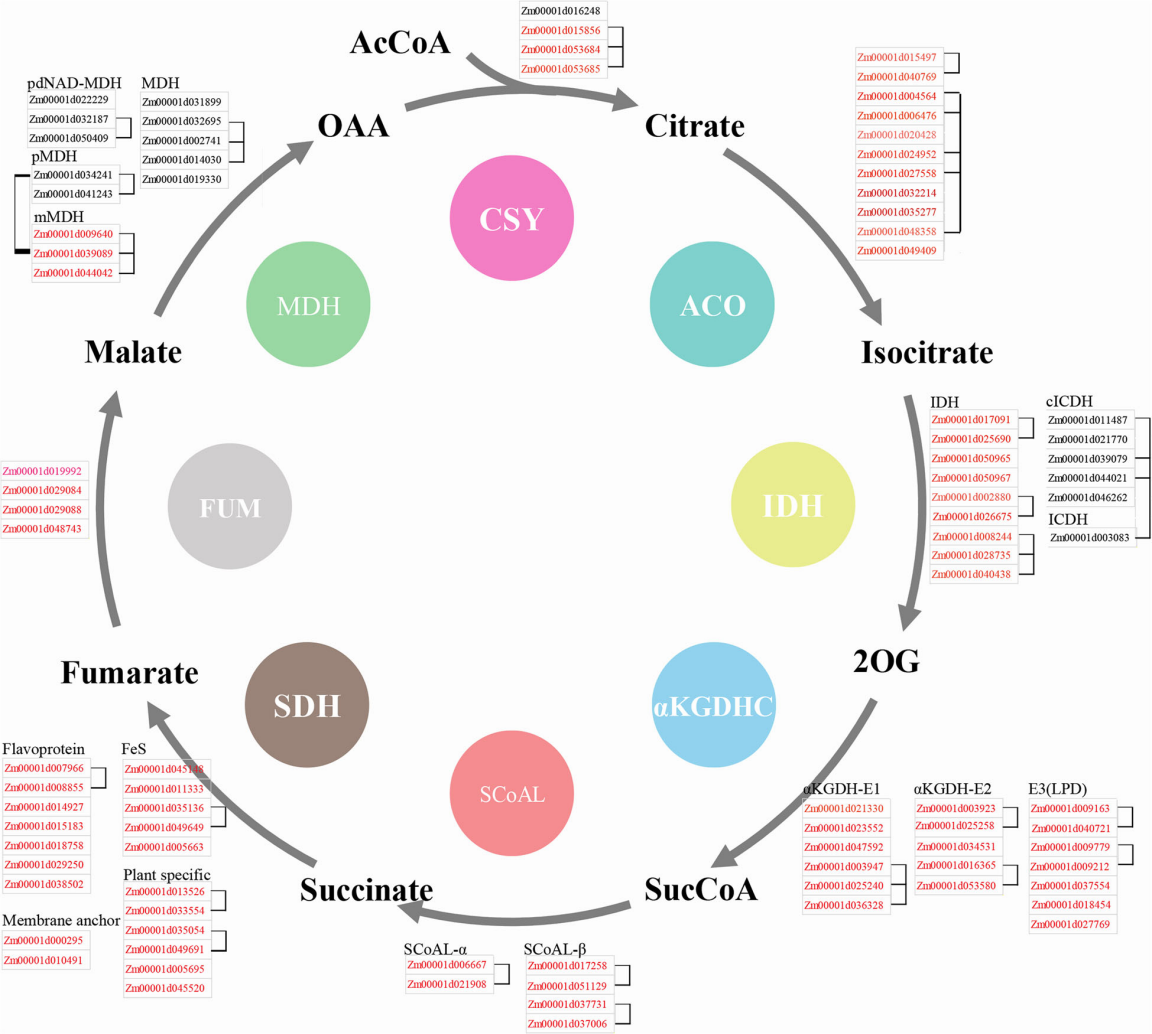
Distribution of genes related to different TCA components and their amino acid sequence similarity analysis. The circle indicates the enzyme complex. The maize TCA genes were anchored to each enzyme complex or subunit based on the highest score in BLASTP against Arabidopsis TCA proteins. The two linked genes indicate that their aligned amino acid sequences account for more than 70% of the longer protein, and the identity of the aligned regions is greater than 70%. Proteins localized in mitochondria are shown in red font, and the localization information for all TCA proteins is derived from homologous proteins in Arabidopsis (Cavalcanti et al., 2014)

**Fig. 2:**
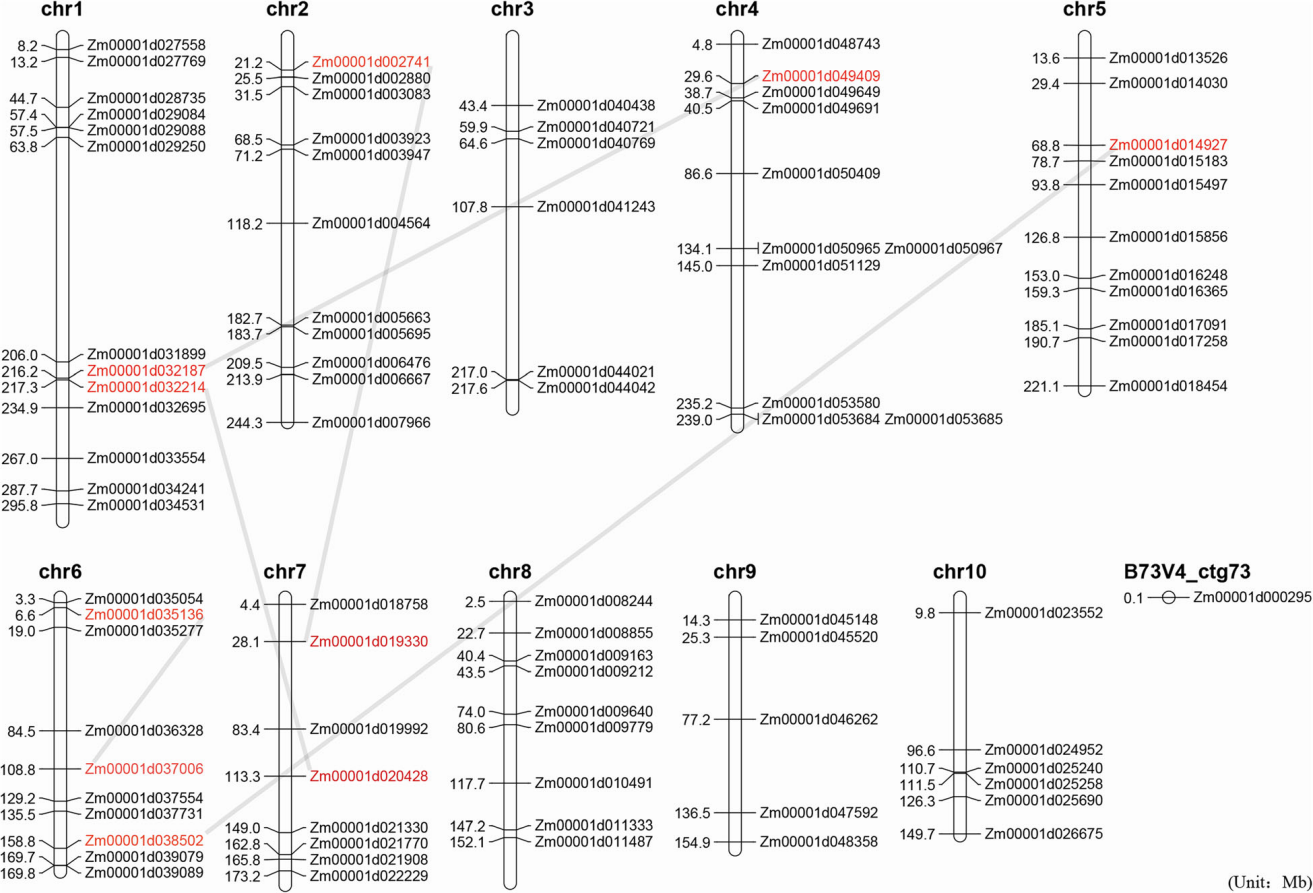
Distribution and duplication event analyses of the maize TCA cycle genes. The location of each TCA gene is shown to the right of each chromosome. Several genes highlighted in red and joined by lines are the product of gene duplication

To comprehensively analyse the functions of the maize TCA genes, 91 TCA cycle genes were identified in the maize genome through sequence similarity searching, and few genes with higher E-values than the threshold were also included in this study (Fig. 1 and Additional file 2). For the eight TCA enzymes, 20 SDH genes, 18 αKGDHC genes, and 15 IDH genes were identified. In addition, four genes were identified for both CSY and MDH, and 13, 11 and 6 genes were identified for MDH, ACO, and SCoAL, respectively. At the same time, we found that genes located in the same enzyme or subunit tended to share high protein sequence similarity (Fig. 1). Mapping all the TCA genes to the maize genome showed that they were unevenly distributed on 10 chromosomes, and Zm00001d000295 was on a contig. Gene duplication analysis showed that there were five pairs of duplicate genes among the maize TCA cycle genes (Fig. 2), and each pair of duplicate genes was always from the same enzyme complex, indicating that gene duplication events play an important role in maize TCA gene expansion.

### Phylogenetic analysis of TCA proteins in maize, Arabidopsis and tomato

**Fig. 3:**
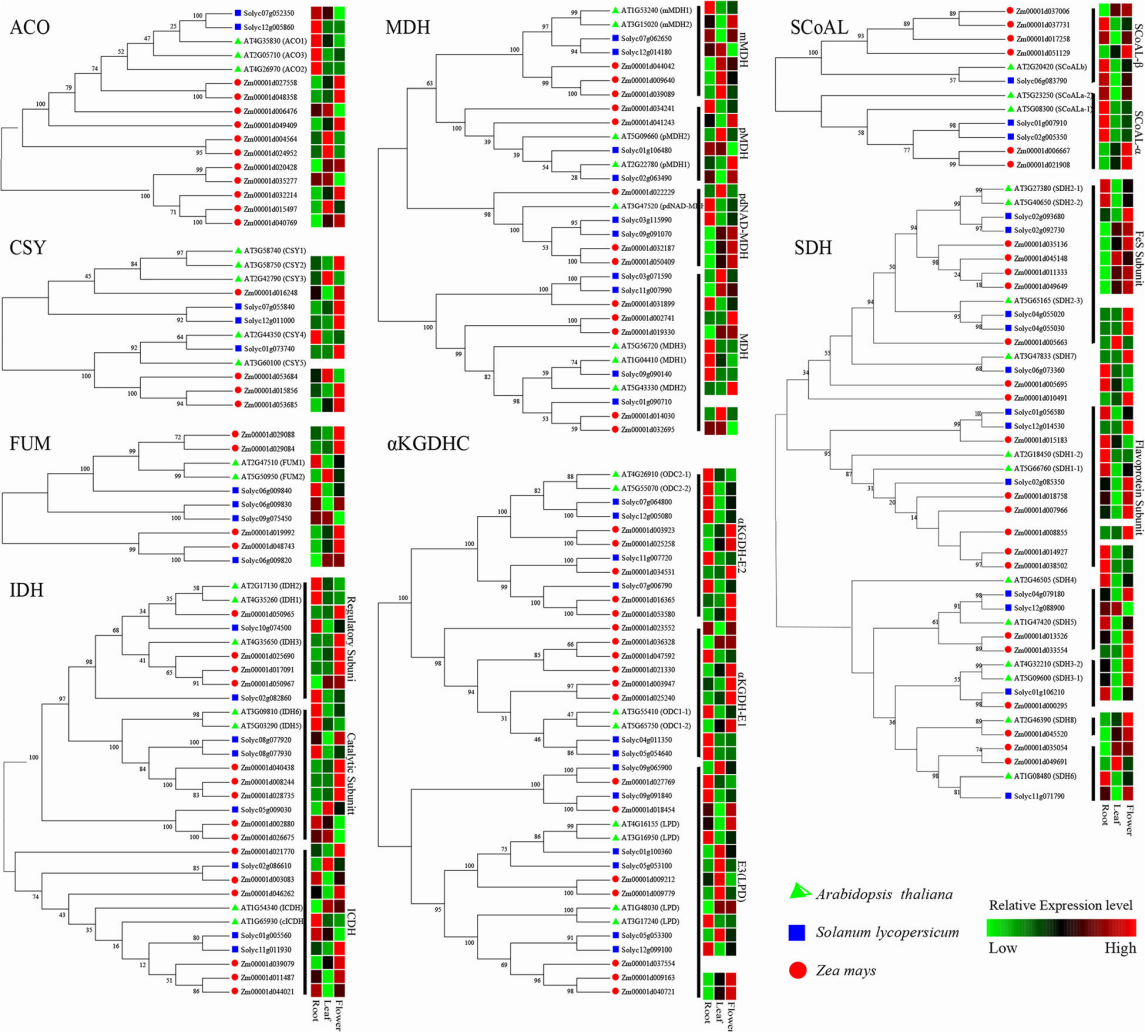
Phylogeny and expression analysis of the TCA cycle genes among maize (*Zea mays*), Arabidopsis (*Arabidopsis thaliana*) and tomato (*Solanum lycopersicum*). Each phylogenetic tree was constructed by MEGA5.0 using the neighbour-joining method with 1000 bootstrap values, and the phylogenetic tree of the SDH proteins was constructed by the maximum likelihood estimate method. The gene expression data were downloaded from TRAVA (http://travadb.org/, Arabidopsis), Tomexpress (http://tomexpress.toulouse.inra.fr/, tomato), and qTeller (www.qteller.com, maize). The expression characteristics of each gene were evaluated by the Z-score

Given that more information was available regarding the molecular functions of the Arabidopsis and tomato TCA genes, a series of phylogenetic trees of TCA proteins among maize, Arabidopsis and tomato was constructed (Fig. 3). The results showed that the 194 TCA proteins were categorized into 8 groups according to the TCA enzyme complexes. Additionally, due to the presence of enzyme subunits, those proteins from IDH, αKGDHC, and SDH were divided into three subgroups: SCoAL had two subgroups, and MDH proteins encompassed four subgroups. Based on the phylogenetic analysis, numerous paralogous proteins were found, including ACO (4 pairs), CSY (1 pair), FUM (1 pair), IDH (4 pairs), MDH (4 pairs), αKGDHC (6 pairs), SCoAL (2 pairs), and SDH (3 pairs). The presence of extensive paralogous pairs among TCA proteins indicated that the expansion of maize TCA genes mainly occurred after the division of paralogous genes. Interestingly, several distinct branches in the ACO, IDH, MDH, αKGDHC, SCoAL, and SDH subfamilies only consisted of maize proteins, suggesting that some maize TCA members were expanded by gene duplication.

### Analysis of the promoter regions of the maize TCA genes

**Fig. 4:**
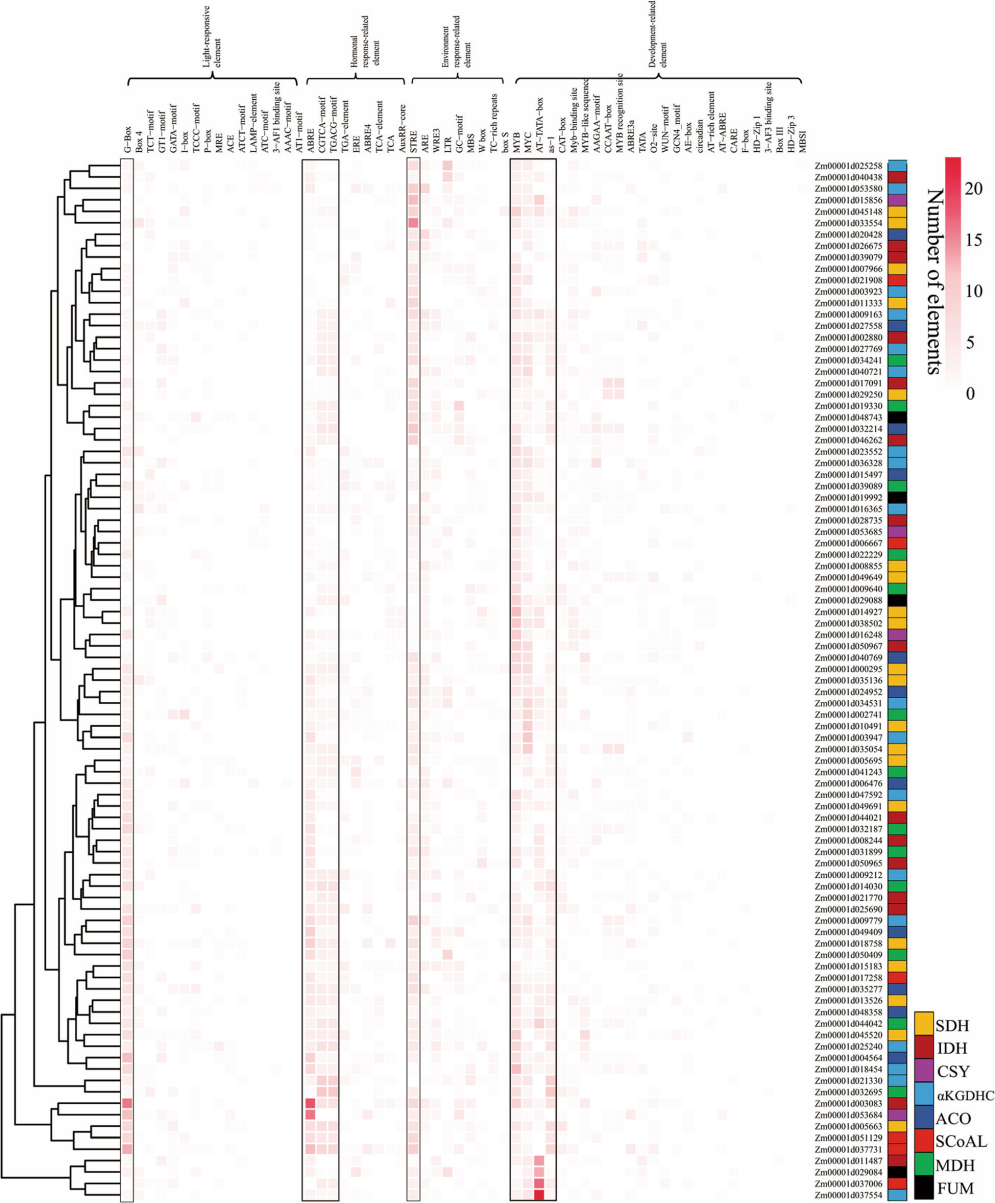
Promoter analysis of the maize TCA cycle genes. Sequences (2000 bp) upstream of the transcriptional start sites of each maize TCA gene were submitted as a promoter to PlantCARE (http://bioinformatics.psb.ugent.be/webtools/plantcare/html/) for cis-acting element analysis. Genes were clustered based on their promoter elements, and those elements appearing more than 200 times in all maize TCA gene promoters were circled

The cis-acting elements of each gene promoter region were analysed to uncover the expression patterns of the maize TCA cycle genes. Several regulatory elements related to important physiological processes, such as light-responsive, hormonal-response, environmental-response, and development-related elements were found in their promoter regions (Fig. 4). The light-responsive element G-box was widely present in the promoter region of each gene, indicating that these genes might be involved in photosynthesis and carbohydrate metabolism. ABA and MeJA are important signalling molecules for the plant stress response, and their corresponding response elements, ABRE (ACGT-containing abscisic acid responsive element) [38], CGTCA-motif (MeJA-responsive element) [39], and TGACG-motif (MeJA-responsive element) [39], were also present in the promoter regions of multiple TCA genes, indicating that these genes could be involved in biotic or non-biotic stress responses. Additionally, the promoters of several TCA cycle genes contained many environmental response-related elements, such as STRE (stress-responsive element) [40] and LTR (low-temperature responsive element) [41], suggesting that the expression levels of these genes might be regulated by ambient pressure. Furthermore, many MYB and MYC recognition and binding elements were found in the promoters, implying that their expression may be regulated by MYB and MYC transcription factors. In addition, we also found that the cis-acting elements of some genes in the same enzyme complexes were similar, including Zm00001d007966 and Zm00001d013966 (SDH), Zm00001d021908 and Zm00001d00667 (SCoALɑ), Zm00001d009640 and Zm00001d039089 (MDH), Zm00001d040721 and Zm00001d025258 (ɑKGDH), Zm00001d008244 and Zm00001d025690 (IDH). This suggested that they might collaborate during plant development.

### Expression profiles of the maize TCA cycle genes

First, the expression levels of the TCA genes in Arabidopsis, tomato and maize were compared (Fig. 3): (1) most of the CSY, aKGDHC, IDH, and MDH genes were expressed in the roots, leaves and flowers of all three species; (2) the expression levels of most SDH genes were low in the leaves but high in the roots and flowers; (3) most of the genes of ACO and SCoAL were specifically expressed in the roots of Arabidopsis and tomato but distinctively expressed in the leaves and tassels of maize; (4) the FUM genes were expressed in the roots, leaves, and flowers of Arabidopsis and tomato but were particularly expressed in the tassels of maize.

**Fig. 5:**
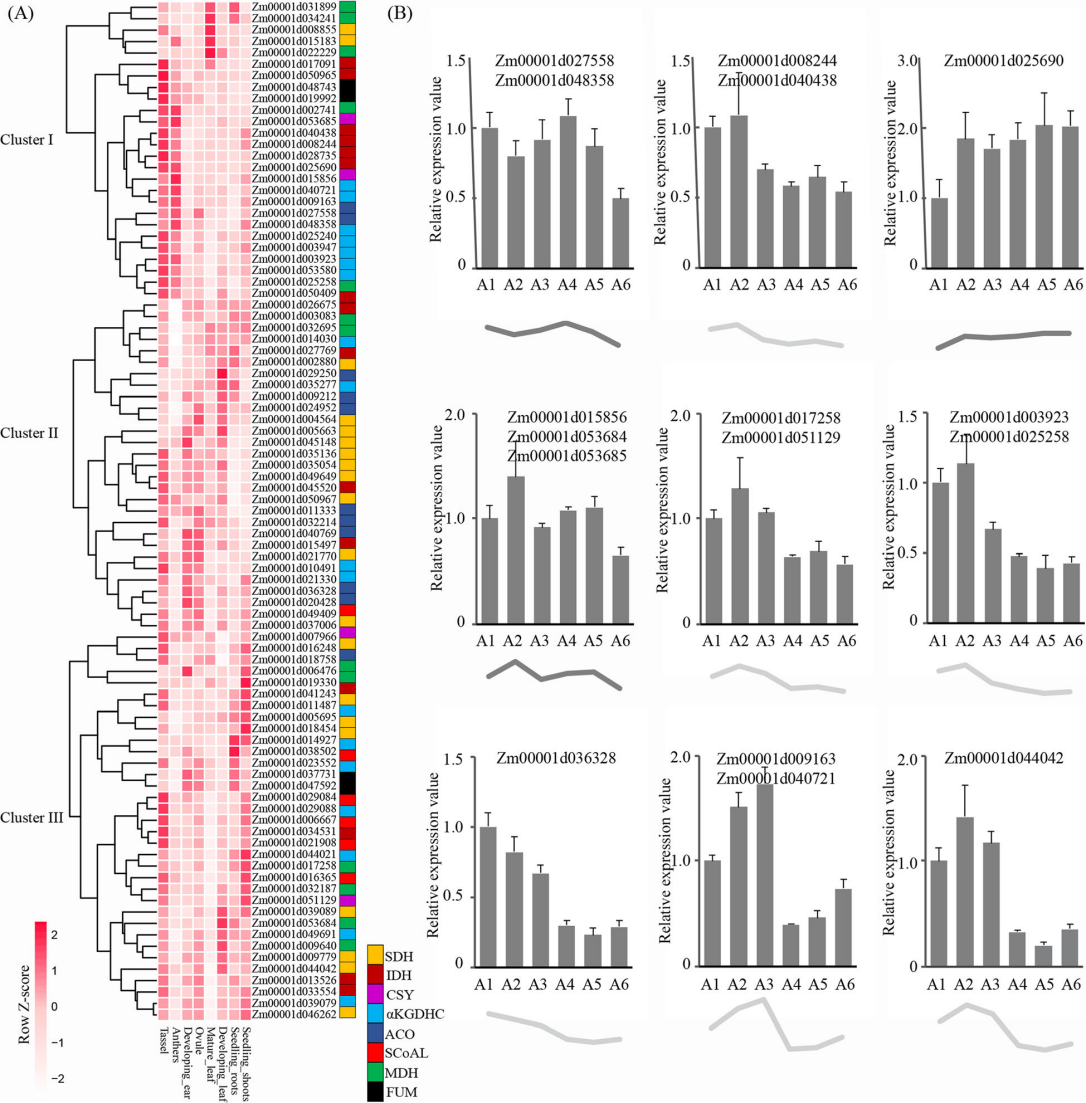
Expression profiles of maize TCA genes. **a** The expression levels of TCA genes in different organs. The gene expression data were retrieved from qTeller (http://www.qteller.com). **b** qPCR analysis of maize TCA genes in the anthers. A1, A2, A3, A4, A5 and A6 denote developing anthers with lengths of 1.0~1.5 mm (cell differentiation), 1.5~2.0 mm (meiosis I), 2.0~2.5 mm (meiosis II) and 2.5~3.0 mm (uninucleate microspore), 3.0~3.5 mm (early binucleate microspore), and 3.5~4.0 mm (late binucleate microspore), respectively. The data are given as the means ± SEMs of at least three biological replicates

Next, the expression patterns of the maize TCA cycle genes in different organs were investigated. We found that the maize TCA genes were widely expressed in almost all of the tested organs and could be divided into 3 subgroups according to their expression patterns (Fig. 5a): (1) the first cluster of genes was mainly expressed in the anthers and tassels; (2) most of the genes belonging to the second cluster were expressed in various organs; and (3) the remaining genes in the third group were principally expressed in the tassels and shoots. At the same time, the expression profile showed that most of the TCA genes were preferentially expressed in the anthers and tassels, which might be caused by the substantial energy demand of the male reproductive organs. Interestingly, it seemed that the genes in the same enzyme complex always shared a similar expression profile, indicating that those duplicate genes with similar expression patterns might be functionally redundant and might have retained their function during evolution.

Further, for the maize TCA genes with high expression in male reproductive organs, we used qPCR to analyse their expression levels in the different stages of microspore development (Fig. 5b). It was found that the expression characteristics of these TCA genes in the anthers could be divided into two categories: (1) the expression levels of some genes in different microspore developmental stages appeared to be relatively stable, such as Zm00001d027558, Zm00001d048358, Zm00001d025690, Zm00001d015856, Zm00001d053684, and Zm00001d053685, and (2) the remaining genes showed high expression levels during microspore meiosis but low expression levels during mitosis, such as Zm00001d008244, Zm00001d040438, ZM00001d017258, Zm00001d051129, Zm00001d003923, Zm00001d025258, Zm00001d036328, Zm00001d009163, Zm00001d040721, and Zm00001d044042.

### Subcellular localization analysis

**Fig. 6:**
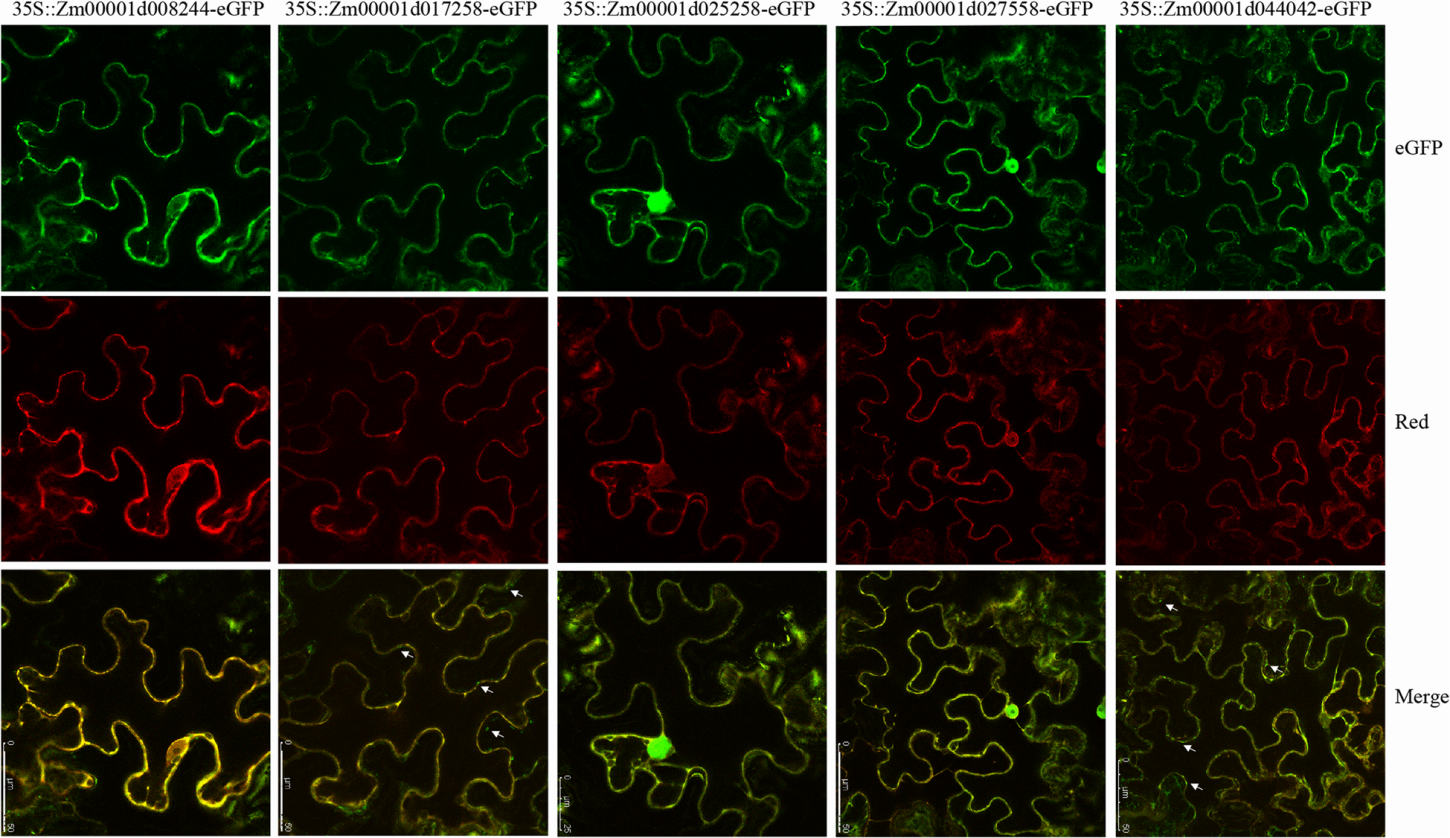
Subcellular localization analysis of maize TCA genes. eGFP indicates the fusion protein, the red fluorescence is the mitochondrial marker, and the yellow colour is the merged result

In this study, all the maize TCA protein localizations were preliminarily predicted based on their Arabidopsis homologue locations [42]. Most TCA proteins were located in mitochondria, but some proteins were localized to the cytosol, peroxisome or chloroplast (Fig. 1 and Addition file 2). Based on the above prediction results, five genes from different enzyme complexes were selected for further subcellular localization analysis in tobacco leaves. As shown in Fig. 6, the mitochondrial marker with red fluorescence was distributed in a dotted pattern in the cell. Moreover, for each gene, most of their green fluorescence overlapped with red fluorescence, suggesting that all five genes were located in mitochondria. As indicated by the arrow in the Fig. 6, we found that Zm00001d017258 (SCoAL) and Zm00001d044042 (MDH) were also in other locations of the cytoplasm. In the leaves transformed with Zm00001d025258 (αKGDH) and Zm00001d027558 (ACO), we found that some green fluorescence also appeared in the nucleus, indicating that these two genes were located in both the mitochondria and nucleus.

### Interaction networks of the maize TCA genes

**Fig. 7:**
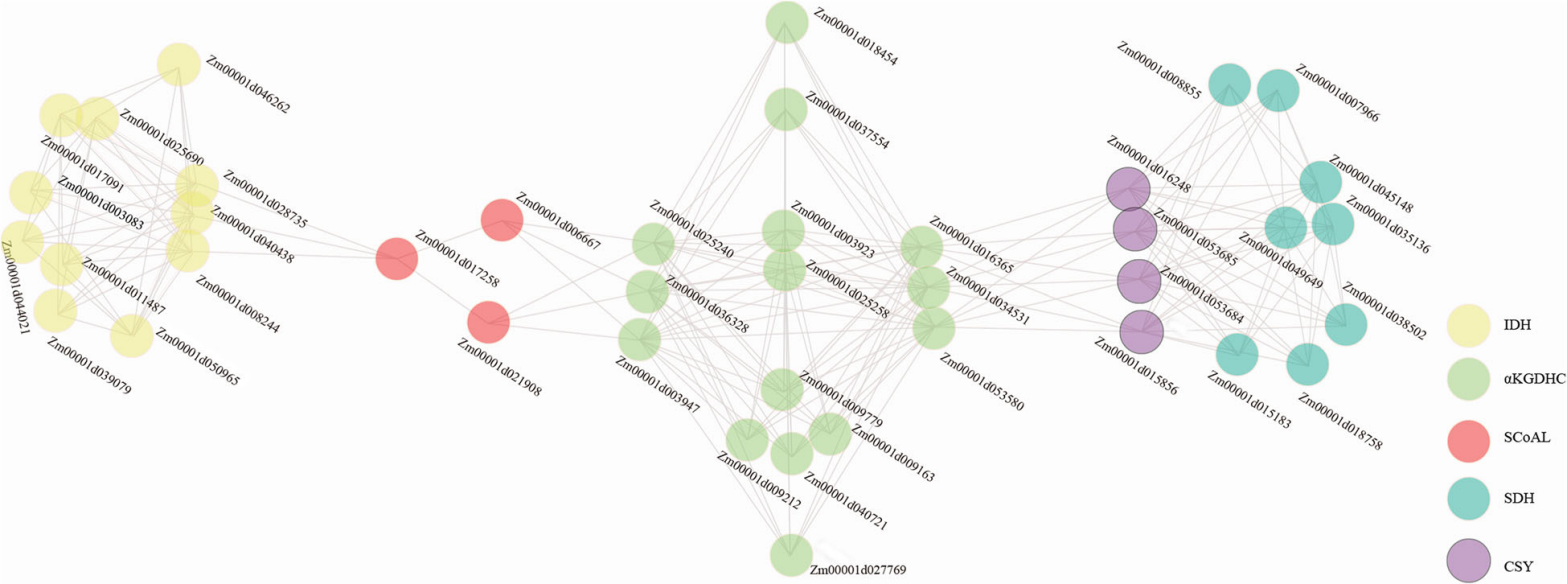
Interaction network prediction of maize TCA genes. All maize TCA genes were submitted to STRING (https://string-db.org) to construct their interaction networks. Only those interactions verified in other species are shown

**Fig. 8:**
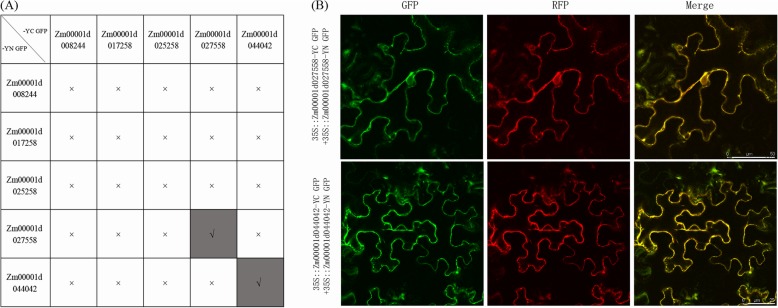
Interaction analysis of five maize TCA genes using BiFC. **a** Summary of interactions among the five TCA cycle genes. “√” indicates an interaction, and “×” indicates no interaction. **b** Homodimers observation of Zm00001d027558 and Zm00001d044042. eGFP indicates the fusion protein, the red fluorescence is the mitochondrial marker, and the yellow colour is the merged result

Protein interactions in the plant TCA cycle are critical for intracellular enzymatic reactions. Herein, we constructed the interaction networks among all maize TCA genes using STRING software (Fig. 7). A total of 41 proteins from 5 enzyme complexes participated in the TCA interaction networks, among which 15 genes from the aKGDHC complex participated in the interaction, while ACO, MDH and FUM genes did not participate in the interaction networks. Based on the predicted results, we found that the interaction between the maize TCA genes occurred mainly between those proteins from the same enzyme or the same subunit. Further, a total of 25 sets of protein interaction tests were performed through BiFC to investigate the interaction among the above five genes (Fig. 8a). It was found that in all 25 groups of protein interaction tests, only Zm00001d027558 (ACO) and Zm00001d044042 (MDH) formed homodimers, and no heterodimers were observed (Fig. 8b). In addition, previous protein localization results indicated that Zm00001d027558 (ACO) was localized in both the mitochondria and nucleus, while the BiFC result showed that its homodimer only formed in the mitochondria. Similarly, Zm00001d044042 (MDH) was localized to the mitochondria and cytoplasm, but its homodimer was only in the mitochondria.

### Root length analysis of the Arabidopsis lines overexpressing five maize TCA genes

**Fig. 9:**
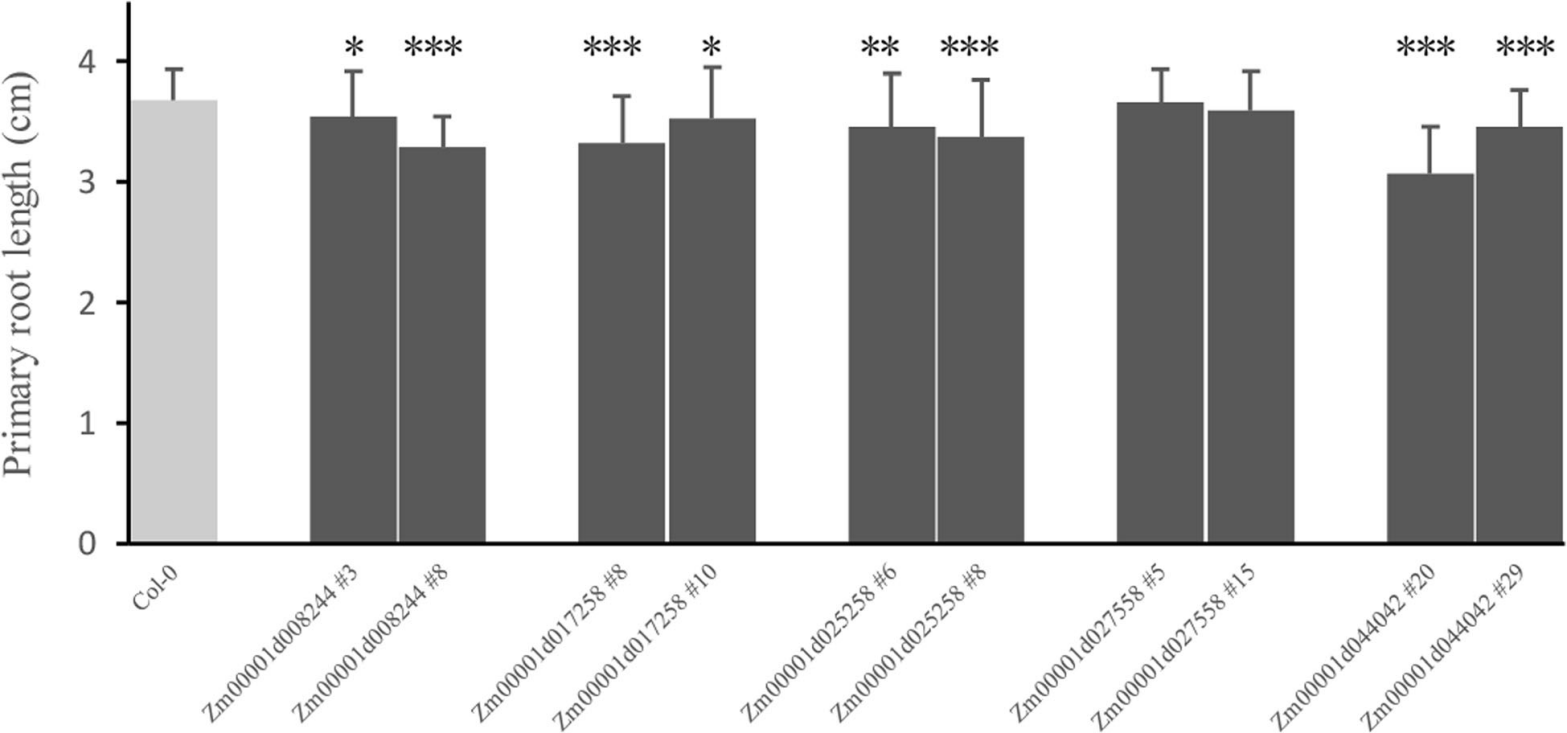
Primary root length analysis of the Arabidopsis overexpression lines. The data are presented as the mean ± SD of 20 seedlings, and three independent tests were performed. The asterisks represent statistically significant differences between the overexpression lines and wild type (Col-0) at *P* < 0.05 (*), *P* < 0.01 (**) or *P* < 0.001 (***) by ANOVA

In this study, five TCA genes, Zm00001d008244 (IDH), Zm00001d017258 (SCoAL), Zm00001d025258 (ɑKGDH), Zm00001d027558 (ACO), and Zm00001d044042 (MDH), were overexpressed in Arabidopsis for functional studies. The five genes were selected for functional studies mainly because they came from different enzyme complexes and had different expression patterns, and the analysis of their functions could reflect the functional diversity of the TCA cycle genes. A series of overexpressed single-copy Arabidopsis lines for the five TCA genes were identified through antibiotic screening, PCR, and confocal microscopy (Additional file 3). Among them, Zm00001d008244 (#3, #8), Zm00001d017258 (#8, #10), Zm00001d025258 (#6, #8), Zm00001d027558 (#5, #15), and Zm00001d044042 (#20, #29) were selected for further phenotypic analysis. As shown in Fig. 9, the lengths of the primary roots of the overexpression lines for Zm00001d008244 (IDH), Zm00001d017258 (SCoAL), Zm00001d025258 (ɑKGDH), and Zm00001d044042 (MDH) were significantly shorter than those of the wild type, while the primary root lengths were nearly unaltered when Zm00001d027558 (ACO) was overexpressed. The above results suggested that a few TCA genes were associated with root development. However, it seemed that the changes in primary root length did not have an apparent effect on plant growth because no obvious changes in shoot and overall growth were observed between the overexpression plants and the wild type.

### Salt stress analysis of the Arabidopsis lines overexpressing five maize TCA genes

**Fig. 10:**
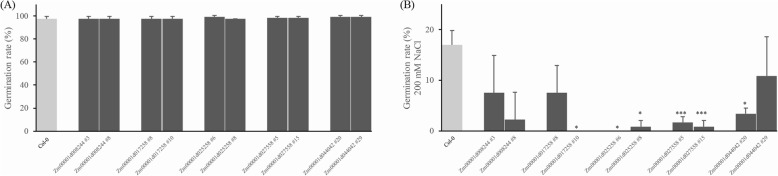
Germination rate of the Arabidopsis overexpression lines under normal conditions (**a**) and 200 mM NaCl (**b**). The data are presented as the mean ± SD of 40 seeds, and three independent tests were performed. The asterisks represent statistically significant differences between the overexpression lines and wild type (Col-0) at *P* < 0.05 (*) or *P* < 0.001 (***) by ANOVA

In normal 1/2 MS medium, the germination rate of each overexpressed line was more than 97%, which was not different from the wild type germination rate (Fig. 10a). Under the stress of 200 mM NaCl, the germination rate of wild type was approximately 17%, and that of the overexpression lines was generally less than 10% (Fig. 10b). Among them, the germination rate of the transgenic lines of Zm00001d025258 and Zm00001d027558 was even less than 2%, which was significantly lower than that of wild type. The above results indicated that overexpression of the candidate genes Zm00001d025258 (ɑKGDH-E2) and Zm00001d027558 (ACO) could increase the sensitivity of Arabidopsis to salt.

### Fertility analysis of the Arabidopsis lines overexpressing five maize TCA genes

**Fig. 11:**
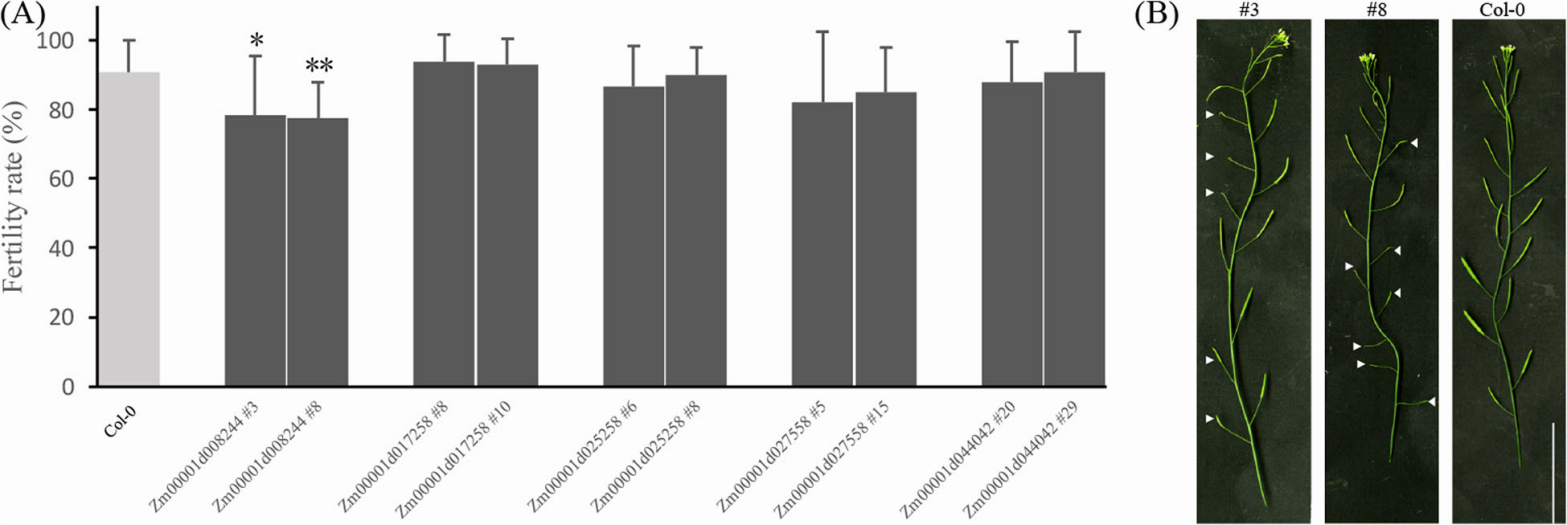
**a** Analysis of the fertility of the Arabidopsis overexpression lines. The data were calculated based on the average of two independent experiments, and each experiment contained 10 plants from each line; the error bars indicate the standard deviation. Differential significance analysis was performed using the ANOVA test; * indicates *P* < 0.05, and ** indicates *P* < 0.01. **b** The overexpression of Zm00001d008244 reduced Arabidopsis fertility. The arrows indicate shorter and empty siliques on the main stem. The ruler is 5 cm

To reveal the role of the TCA cycle in plant reproduction, the silique appearances of the corresponding overexpressed lines were inspected. We found that the silique appearances of the Zm00001d017258-, Zm00001d025258-, Zm00001d027558-, and Zm00001d044042- overexpressing lines were similar to those of the wild type (Fig. 11a). In contrast, the Zm00001d008244 (IDH)-overexpressing line exhibited fertility deficiency compared to the wild type. In particular, few short and empty siliques were observed for the Zm00001d008244-overexpressing lines (Fig. 11b). In addition, the protein abundances of Zm00001d008244 in the overexpression lines were confirmed by western blot (Additional file 4). The above results indicated that the excessive accumulation of the Zm00001d008244 protein reduced the fertility rate of Arabidopsis.

## Discussion

The TCA cycle is ubiquitous in animals, plants and various microorganisms, and it can produce NADH for oxidative phosphorylation and organic acids required for biosynthesis, such as oxaloacetate acid, citric acid, fumaric acid and malic acid [1, 3, 20]. In this study, we identified 91 genes that make up the TCA cycle enzyme complexes of maize. Among them, five genes (Zm00001d008244 (IDH), Zm00001d017258 (SCoAL), Zm00001d025258 (ɑKGDH), Zm00001d027558 (ACO), and Zm00001d044042 (MDH)) were selected for further functional analysis, which were distributed on five different enzyme complexes. Subcellular localization results showed that the five proteins were mainly located in the mitochondria, suggesting that they mainly function in the mitochondria. However, Zm00001d025258 and Zm00001d027558 also appeared in the nucleus, and the translational products of Zm00001d017258 and Zm00001d044042 were distributed in the cytoplasm, suggesting that the above four genes might have other functions in the cell. Some TCA cycle genes played a role in multiple cellular locations in addition to the mitochondria, which reflects the diversity of TCA genes [2].

In the Arabidopsis TCA cycle, a large number of interactions were found, including those between catalytic subunits of the same or different enzymes [43]. In this study, we predicted many interactions among maize TCA genes, but this requires further experimental verification. Further, through BiFC, we did not detect any heterodimers between the above five TCA genes, and only found that Zm00001d027558 (ACO) and Zm00001d044042 (MDH) could form homologous dimers. Interestingly, these dimers were present only in the mitochondria, which was not entirely consistent with their protein localization. In addition to the mitochondria, the proteins Zm00001d027558 and Zm00001d044042 were located in the nucleus and cytoplasm, respectively. Previously, it was found that heterologous dimerization [44, 45], alternative splicing [46], and external signal stimulation [47, 48] could change protein cellular localization. In the present study, our results showed that the homologous dimerization of two proteins, Zm00001d027558 and Zm00001d044042, changed their intracellular localization, but the exact significance remains to be resolved.

In this study, the above five TCA genes were overexpressed in Arabidopsis to reveal their potential roles in plant development. Studies have shown that tricarboxylic acid cycle genes are closely related to root development. For example, in tomato, the inhibition of MDH, ACO or FUM can reduce root respiration activity and dry matter accumulation [15–18]. In the present study, we found that the overexpression of the three genes Zm00001d008244 (IDH), Zm00001d017258 (SCoAL), and Zm00001d044042 (MDH) significantly shortened the length of the primary root of Arabidopsis. The overexpression of some energy-related genes could lead to mitochondrial dysfunction, which ultimately affects plant normal development [49–51]. For our research results, we hypothesized that elevated expression levels of these TCA cycle genes might cause certain negative effects on mitochondria and ultimately inhibit root growth.

The coordinated expression of energy metabolism genes is critical for the growth and development of plants under stress conditions [52]. Photosynthesis is the principal energy source of cells for growth under favourable conditions, while under stress, the rate of photosynthesis could decrease, and glycolysis and the TCA cycle would provide energy to cells [53]. As a key part of energy metabolism, the TCA cycle has been proved to be involved in maize salt tolerance [36, 37, 54]. In this experiment, the overexpression of Zm00001d025258 (ɑKGDH) and Zm00001d027558 (ACO) enhanced the sensitivity of Arabidopsis to NaCl stress, indicating their application value in plant stress resistance. Moreover, plant seeds would dodge to avoid environmental stress, thus reducing the seed germination rates under stress conditions [55]. For example, AtFUSCA3 delays seed germination under high-temperature conditions, but restores seed germination at an appropriate temperature [56]. Therefore, Zm00001d025258 (ɑKGDH) and Zm00001d027558 (ACO) might participate in plant stress resistance by inhibiting seed germination under stress.

The TCA cycle is closely related to plant flower development. For example, the inhibition of pyruvate dehydrogenase E1a subunit expression in tobacco could cause male sterility [9], dysfunctional SDH could lead to abnormal gametophyte development, aborted pollen and decreased seed quantity in Arabidopsis [10]. Isocitrate dehydrogenase (IDH) oxidizes isocitrate to form ɑ-ketone glutaric acid. In this study, we found that the overexpression of the maize IDH gene Zm00001d008244 could reduce plant fertility. In the future, it will be necessary to check whether male gametes, female gametes or embryo development cause fertility deficiency. Surprisingly, there were no significant phenotypic changes in the IDH knockout mutant of Arabidopsis and the IDH antisense mutant of tomato [19, 57, 58]. We speculated that this might be due to functional differences in the TCA genes among different species. For instance, in soybean, the GmmMDH1 null mutant turned green leaves to yellow [59], while GhmMDH1 knockdown in cotton resulted in decreased malic acid content, increased respiration rate and decreased biomass [60]. However, the inhibition of mitochondrial malate dehydrogenase in tomato could decrease the respiration rate of roots and leaves but increase the dry matter mass of aboveground parts and fruit size and induce an earlier flowering period [16, 18]. In Arabidopsis, the mitochondrial malate dehydrogenase T-DNA double mutant mmdh1mmdh2 exhibited smaller and slower growth and had an increased leaf respiration rate [61]. In this experiment, except for Zm00001d008244, the other genes did not influence plant fertility. The TCA cycle is associated with the circadian clock, which regulates plant reproductive development [62–64], so it would be interesting to determine whether changes in circadian rhythm affect the fertility of those overexpression plants. In summary, the above results reflect the functional diversity of the TCA genes among different species.

## Conclusions

In conclusion, 91 genes were identified in the maize TCA cycle, and these genes were distributed on eight enzyme complexes. The chromosomal locations, duplication events, polygenetic relationships, expression patterns, subcellular localizations, and interaction networks of all the maize TCA cycle genes were determined. Importantly, through a series of phenotypic analyses of five TCA cycle genes overexpressed in Arabidopsis plants, we found that the maize TCA cycle is involved in root development, salt stress, and plant fertility.

## Methods

### Plant materials

All the plant materials used in the present study were originated form our laboratory and experimental research on plants were performed according to the guidelines of the Maize Research Institute of Sichuan Agricultural University (No.211 Huimin Road, Wenjiang District, Chengdu, China). Yongming Liu undertook the formal identification of the samples.

### Bioinformatics analyses of the maize TCA cycle genes

A total of 48 genes in Arabidopsis are directly involved in the TCA cycle [42]; herein, their protein sequences were used as queries to identify TCA genes in maize and tomato using BLASTP (e-value ≤1e-30). Arabidopsis protein sequences were retrieved from GRAMENE (http://www.gramene.org/, TAIR10), maize protein sequences were downloaded from MAIZEGDB (https://www.maizegdb.org/, AGP V4.0), and tomato protein sequences were downloaded from SGN (https://solgenomics.net/, ITAG4.0). A phylogenetic tree was constructed based on the maize, tomato and Arabidopsis TCA proteins by MEGA 5.10. Subsequently, we analysed duplication events among maize TCA genes, and a gene duplication event was defined according to the two following criteria [65]: (1) the alignable nucleotide sequence covers > 70% of the longer gene and (2) the region of identity between them encompasses > 70% of the alignable region. For cis-element enrichment analysis, 2000 bp sequences upstream of the transcription start site of each gene were obtained from the maize genome database (http://ensembl.gramene.org/Zea_mays/Info/Index) and were then submitted to PlantCARE (http://bioinformatics.psb.ugent.be/webtools/plantcare/html/). The genome and promoter sequences of the maize TCA genes were downloaded from GRAMENE (http://www.gramene.org/, B73 RefGen_V4).

### Expression pattern analysis

The expression levels of the TCA genes in the roots, leaves, and flowers (tassels) were compared among Arabidopsis, tomato and maize. The expression data of Arabidopsis, tomato, and maize genes were obtained from TRAVA (http://travadb.org/), Tomexpress (http://tomexpress.toulouse.inra.fr/), and qTeller (www.qteller.com). Subsequently, the expression patterns of the maize TCA genes in various tissues were analysed in detail. Further, qPCR was used to analyse TCA gene expression levels in developing anthers of the maize inbred line Huangzaosi. Total RNA was extracted using TRIzol reagent, and cDNA was synthesized by the PrimeScript RT Reagent Kit (TaKaRa, China) according to the manufacturer’s instructions. The 18S reference gene was used for normalization. All the primers in this study are shown in Additional file 1. qPCR was performed with a CFX96™ Real Time system (Bio-Rad, USA) using SYBR Green Real Time PCR Master Mix (TaKaRa). The relative expression level of each gene was evaluated by the 2^-ΔΔCt^ method [66]. The expression data of each gene were obtained from three biological replicates, each of which was measured at least three times. In addition, part of the expression data was retrieved from our previous report [67].

### Subcellular localization analysis

Five TCA genes (Zm00001d008244, Zm00001d017258, Zm00001d025258, Zm00001d027558, and Zm00001d044042) were cloned and transiently overexpressed in tobacco (*Nicotiana benthamiana*) leaves for subcellular localization analysis. The above five genes were amplified from the maize inbred line B102 by high-fidelity iProof DNA polymerase (Bio-Rad, USA). The amplified fragments were then inserted into the pDONR221 vector by the BP reaction. After sequencing confirmation, all the BP products were fused to the N-terminus of the enhanced green fluorescent protein (eGFP) in the pB7FWG2.0 vector by the LR reaction. Finally, all the recombinants were transformed into Agrobacterium competent cells (C58C1) using the freeze-thaw method and were finally ectopically expressed in tobacco leaves. After the tobacco plants were cultivated in a greenhouse for 3 days, a Leica TCS SP2 laser confocal microscope (Leica, Germany) was used to observe the green (Ex = 488 nm, Em = 500~530 nm) and red (Ex = 561 nm, Em = 600~630 nm) fluorescence. In this experiment, 35S::mts-RFP was used as a mitochondrial marker.

### Interaction analysis of the maize TCA genes

The interaction networks of the maize TCA proteins were constructed using STRING software (https://string-db.org). Then, we analysed the interactions among the five TCA genes (Zm00001d008244, Zm00001d017258, Zm00001d025258, Zm00001d027558, and Zm00001d044042) using BiFC. As stated above, the five genes were first inserted into the entry vector pDONR221 by the Gateway BP reaction, and the expression vectors 35S::CDS-YC GFP (GFP C-terminus) and 35S::CDS-YN GFP (GFP N-terminus) were constructed by the LR reaction. The recombinant plasmids were transformed into the competent Agrobacterium C58C1 cells, and the leaves of *Nicotiana benthamiana* were infested for fluorescence observation. The detailed steps were the same as those above.

### Generation of TCA gene-overexpressing Arabidopsis plants and phenotypic analysis

To analyse the molecular function of the TCA cycle genes in plant developmental processes, the above fives genes were respectively overexpressed in Arabidopsis. First, all the 35S::CDS-eGFP expression vectors were constructed as above. Second, all the constructed expression vectors were respectively transformed into Agrobacterium C58C1 with the freeze-thaw method and infiltrated into Arabidopsis (Col-0) inflorescence. The positive transgenic plants were simultaneously screened using an antibiotic (50 μg/mL BASTA) on 1/2 MS medium and PCR. The primers for PCR identification were attB1F (5′-GGGGACAAGTTTGTACAAAAAAGCAGGC-3′) and attB2R (5′-ACCCAGCTTTCTTGTACAAAGTGGTCCCC-3′), and the Arabidopsis housekeeping gene *EF1α* (F: 5′-GGCTGCTGAGATGAACAA-3′, R: 5′-GTGGTGGAGTCAATGATAAG-3′) was used as a positive control for PCR amplification. In addition, the green fluorescence intensity in the leaves and main roots of transgenic Arabidopsis were detected to determine highly expressed lines. Finally, the homologous and single copy T_3_ overexpressed lines were chosen for phenotypic analysis.

In this study, the primary root length, salt tolerance, and fertility of the overexpression Arabidopsis were analysed. For the primary root length analysis, 20 seeds for each line were first placed on 1/2 MS medium and then vernalized at 4 °C for 3 days. Thereafter, all the petri dishes were placed vertically in a growth chamber with a 16 h light/8 h dark cycle, and the length of the primary roots was measured after being cultured at 23 °C for 9 days; three independent tests were performed for the root length analysis. For salt tolerance analysis of transgenic lines, 40 seeds of each line were planted on 1/2 MS and 1/2 MS 200 mM NaCl media respectively. After vernalization, germination rates for each line were counted after the plates were placed vertically in a growth chamber for 9 days. Three independent experiments were performed for the salt tolerance analysis. For analysis of transgenic line fertility, the developed siliques on the main stem were examined when the plants were grown in a greenhouse at 23 °C for 40 days. Two independent experiments were conducted for plant fertility investigation. The wild type (Col-0) was used as a control for all the above phenotypic observations.

### Western blot analysis

The western blot method [68] was used to examine the protein levels of Zm00001d008244 in the Arabidopsis overexpression lines. Total protein was extracted from the aboveground part of the overexpression plants in the flowering stage. Anti-GFP rabbit polyclonal antibody (Sangon Biotech, China) was used as the primary antibody, and HRP-conjugated goat anti-rabbit IgG (Sangon Biotech, China) was used as the secondary antibody. The housekeeping protein β-Actin was used as an internal control.

### Statistics and data analysis

Statistical analyses of primary root length, germination rates, and silique fertility were conducted with one-way ANOVA (under homogeneity of variance) or Welch’s ANOVA (under heterogeneity of variance) test [69]. All calculations were performed in SPSS20.0 (IBM, USA).

## Supplementary information

**Additional file 1.** All primers used in this experiment.

**Additional file 2.** Identification and annotations of maize and tomato TCA cycle genes.

**Additional file 3.** PCR identification of the single-copy lines of the T_3_ generation. attB1F and attB2R were used to amplify candidate genes, and the reference gene EF1ɑ was used as a positive control. WT represents the wild type (Col-0).

**Additional file 4.** Western blot detection of the Zm00001d008244 overexpressed Arabidopsis. WT indicates the negative control (Col-0) and Actin was used as the internal control, #3 and #8 were two different overexpressed Arabidopsis lines of Zm00001d008244.

## Data Availability

All data generated or analysed during this study are included in this published article and its supplementary information files.

## Abbreviations

ACO: aconitase
BiFC: bimolecular fluorescence complementation
CSY: citrate synthase
eGFP: enhanced green fluorescent protein
FP subunit: Flavoprotein subunit
FUM: fumarase
IDH: isocitrate dehydrogenase
LTR: low-temperature responsive element
MA subunit: membrane anchor subunit
MDH: malate dehydrogenase
SCoAL: succinyl-CoA synthetase
SDH: succinate dehydrogenase
STRE: stress-responsive element
TCA cycle: tricarboxylic acid cycle
αKGDHC: α-ketoglutarate dehydrogenase complex
