# Supplementary figures and images for: Identification and characterization of the TCA cycle genes in maize
Source: BMC Plant Biol. 2019 Dec 27;19:592. doi: 10.1186/s12870-019-2213-0 (PMC6935159; doi:10.1186/s12870-019-2213-0)

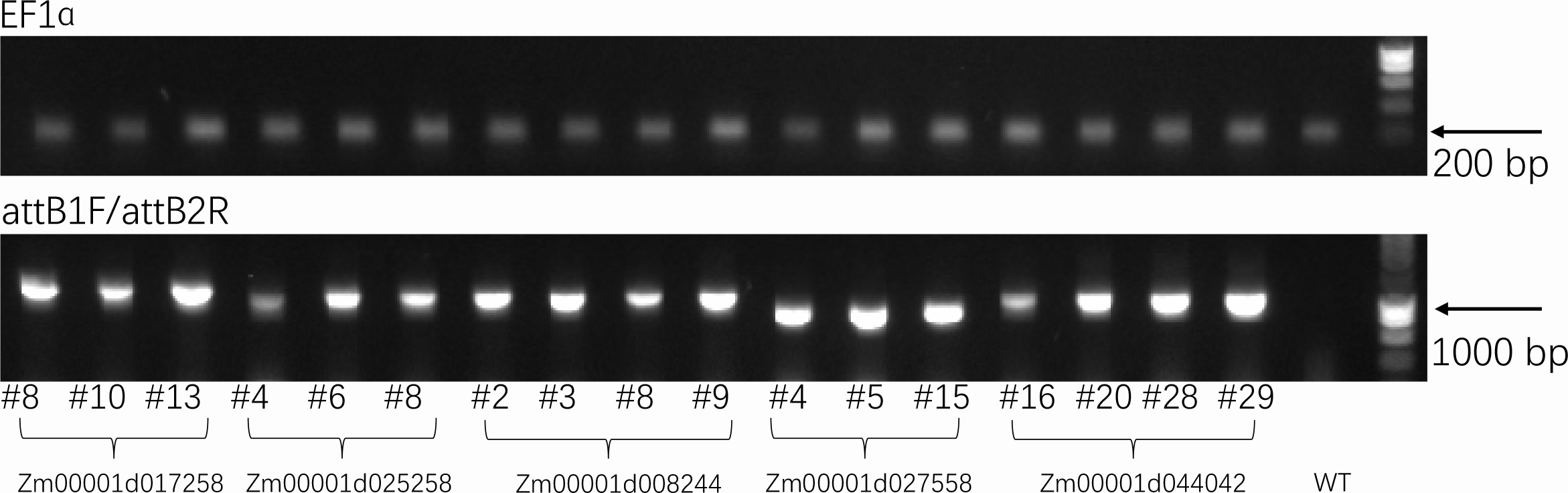

Supplement: Supplementary file 3 — Additional file 3. PCR identification of the single-copy lines of the T3 generation. attB1F and attB2R were used to amplify candidate genes, and the reference gene EF1ɑ was used as a positive control. WT represents the wild type (Col-0). [file 12870_2019_2213_MOESM3_ESM.tif]

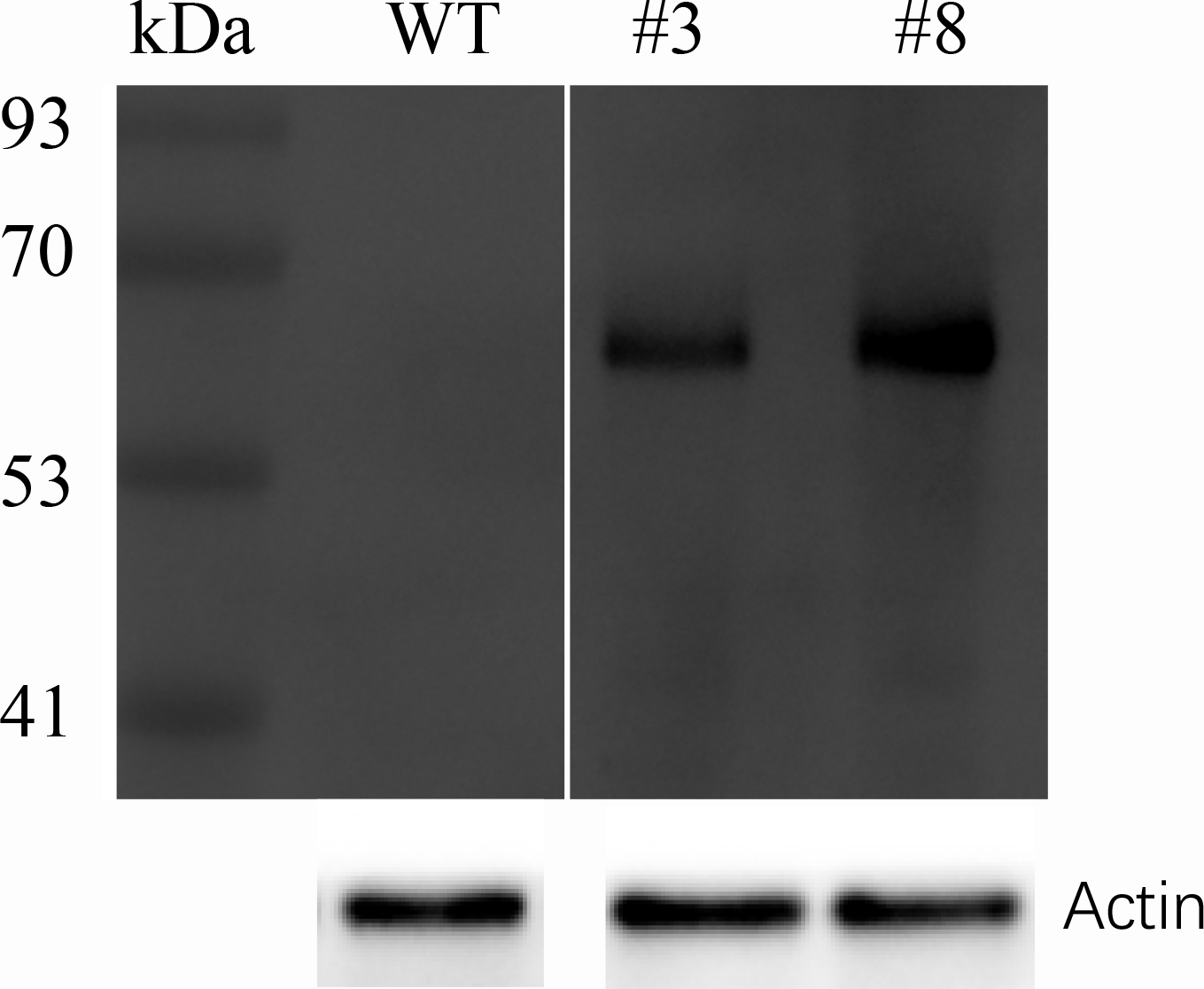

Supplement: Supplementary file 4 — Additional file 4. Western blot detection of the Zm00001d008244 overexpressed Arabidopsis. WT indicates the negative control (Col-0) and Actin was used as the internal control, #3 and #8 were two different overexpressed Arabidopsis lines of Zm00001d008244. [file 12870_2019_2213_MOESM4_ESM.tif]
